# Purine Nucleoside Analog - Sulfinosine Modulates Diverse Mechanisms of Cancer Progression in Multi-Drug Resistant Cancer Cell Lines

**DOI:** 10.1371/journal.pone.0054044

**Published:** 2013-01-11

**Authors:** Mirjana Dačević, Aleksandra Isaković, Ana Podolski-Renić, Andelka M. Isaković, Tijana Stanković, Zorica Milošević, Ljubisav Rakić, Sabera Ruždijić, Milica Pešić

**Affiliations:** 1 Faculty of Medicine, University of Belgrade, Doktora Subotića 8, Belgrade, Serbia; 2 Institute for Biological Research, Department of Neurobiology, University of Belgrade, Bulevar Despota Stefana 142, Belgrade, Serbia; 3 Serbian Academy of Sciences and Arts, Knez Mihailova 35, Belgrade, Serbia; Kyushu University, Japan

## Abstract

Achieving an effective treatment of cancer is difficult, particularly when resistance to conventional chemotherapy is developed. P-glycoprotein (P-gp) activity governs multi-drug resistance (MDR) development in different cancer cell types. Identification of anti-cancer agents with the potential to kill cancer cells and at the same time inhibit MDR is important to intensify the search for novel therapeutic approaches. We examined the effects of sulfinosine (SF), a quite unexplored purine nucleoside analog, in MDR (P-gp over-expressing) non-small cell lung carcinoma (NSCLC) and glioblastoma cell lines (NCI-H460/R and U87-TxR, respectively). SF showed the same efficacy against MDR cancer cell lines and their sensitive counterparts. However, it was non-toxic for normal human keratinocytes (HaCaT). SF induced caspase-dependent apoptotic cell death and autophagy in MDR cancer cells. After SF application, reactive oxygen species (ROS) were generated and glutathione (GSH) concentration was decreased. The expression of key enzyme for GSH synthesis, gamma Glutamyl-cysteine-synthetase (γGCS) was decreased as well as the expression of *gst-π* mRNA. Consequently, SF significantly decreased the expression of *hif-1α*, *mdr1* and *vegf* mRNAs even in hypoxic conditions. SF caused the inhibition of P-gp (coded by *mdr1*) expression and activity. The accumulation of standard chemotherapeutic agent – doxorubicin (DOX) was induced by SF in concentration- and time-dependent manner. The best effect of SF was obtained after 72 h when it attained the effect of known P-gp inhibitors (Dex-verapamil and tariquidar). Accordingly, SF sensitized the resistant cancer cells to DOX in subsequent treatment. Furthermore, SF decreased the experssion of vascular endothelial growth factor (VEGF) on mRNA and protein level and modulated its secretion. In conclusion, the effects on P-gp (implicated in pharmacokinetics and MDR), GSH (implicated in detoxification) and VEGF (implicated in tumor-angiogenesis and progression) qualify SF as multi-potent anti-cancer agent, which use must be considered, in particular for resistant malignancies.

## Introduction

**Figure 1:**
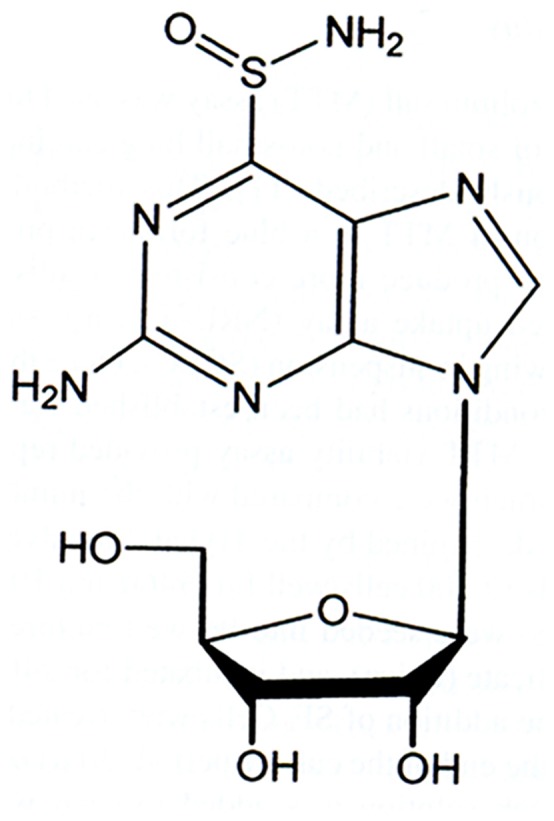
Chemical structure of purine nucleoside analog – sulfinosine (SF).

Sulfinosine or SF (Figure 1, [R,S]-2-amino-9-β-D-ribofuranosylpurine-6-sulfinamide) is the oxidized form of 6-thioguanosine [1]. It is a quite unexplored anti-cancer agent in comparison to other thiopurines (6-thioguanine and 6-mercaptopurine). SF inhibits cancer cell growth, at least partially, by the incorporation of its phosphorylated derivative into DNA. The metabolic conversion of SF to corresponding 5′-monophosphate derivative is more complex than that of other thiopurines [2].

Since SF utilizes different metabolic pathways for its intracellular activation, SF treatment does not induce resistance in cancer cells. In contrast, the deletion of a single enzyme responsible for the metabolic activation of other purine nucleoside analogs is enough for the development of resistance. SF better penetrates the central nervous system (CNS) than its parental molecule - 6-thioguanosine and is more effective in cancer treatment. SF is useful against malignancies resistant to other thiopurines [3]. Despite limitations for their use, some purine analogs closely related to SF showed considerable anti-angiogenic activities that deserve scientific attention [4].

The metabolism of SF involves the cells’ glutathione system. SF readily adducts to sulfhydryl compounds (glutathione and cysteine) and by suppressing the glutathione detoxification system and elevating the concentration of reactive oxygen species (ROS), SF may induce the death of cancer cells [2].

In view of its considerable efficacy in cancer treatment and moderate toxicity to normal cells [2], SF is suitable for combining with other chemotherapeutic agents. SF acts synergistically with doxorubicin (DOX), curcumine (CUR) and verapamil (VER) in non-small cell lung cancer (NSCLC) cell lines [5]–[7]. The effectiveness of the combined application with SF allowed the use of these drugs at lower concentrations that are less toxic with fewer adverse effects. We hypothesized that all mentioned anti-cancer effects of SF could be useful for the reversion of resistance to chemotherapeutics.

Multi-drug resistance (MDR) is the main limitation for the accomplishment of successful cancer treatment. MDR phenotype often relates to the over-expression of P-glycoprotein (P-gp), a membrane transporter that effectively extrudes the cytotoxic drugs from cancer cells and changes their pharmacokinetics. P-gp acts as an efflux pump for various hydrophobic anticancer drugs such as anthracyclines, vinca alkaloids, taxanes, epipodophyllotoxins, and some of the new drugs (e.g. imatinib, nilotinib, everolimus). P-gp over-expression is common in experimental cancer models as well as in cancerous tissues from patients [8]. Therefore, P-gp has become a main therapeutic target for overcoming MDR.

Among many options for reverting MDR, the agents with an anti-cancer activity of their own could be examined as potential MDR modulators. We speculated earlier that besides the synergy between SF and DOX as anti-cancer drugs acting through separate pathways, the alterations of MDR-related genes expression and reduction of P-gp activity could contribute to the chemo-sensitization effect of SF [5], [6].

Therefore, we conducted further investigation of mechanisms involved in SF action in resistant and incurable cancers. For that purpose, we employed two different MDR cancer cell lines with the over-expression of P-gp (NCI-H460/R and U87-TxR) [9], [10]. We studied the potential of SF to kill resistant cancer cells and induce autophagy as well as to modulate the mechanisms involved in cancer progression, such as glutathione (GSH) detoxification system, P-gp mediated drug transport, vascular endothelial growth factor (VEGF) expression and.secretion. We found that the modification of redox status by SF led to the decrease in the expression of hypoxia inducible factor-1α (HIF-1α) which regulates the expression of P-gp and VEGF. Thus, the modulation of MDR by SF is the consequence of GSH detoxification system suppression.

## Materials and Methods

### Drugs

SF ([R,S]-2-amino-9-β-D-ribofuranosylpurine-6-sulfinamide) was synthesized from 6-thioguanosine according to the published procedure [1]. DOX solution was obtained from EBEWE Arzneimittel GmbH, Vienna, Austria. R±Verapamil (Dex-VER) was purchased from Sigma-Aldrich Chemie GmbH, Germany. Tariquidar (TQ) was kindly provided by Dr. Sven Rottenberg from The Netherlands Cancer Institute, Amsterdam. CoCl_2_ was obtained from Fisher Scientific, USA. SF was kept at −20°C. Before treatment, SF and CoCl_2_ were freshly diluted in water, while aliquots of DOX were thawed from −20°C. Dex-VER was kept as 1 mM stock solution at room temperature. TQ was diluted in dimethyl sulfoxide (DMSO) and 10 µM aliquots were kept at −20°C.

### Chemicals

RPMI 1640 medium, Minimum Essential Medium (MEM), penicillin-streptomycin solution, antibiotic-antimycotic solution, L-glutamine and trypsin/EDTA were purchased from PAA, Vienna, Austria. Fetal bovine serum (FBS), sulforhodamine B (SRB) and acridine orange were obtained from Sigma-Aldrich Chemie GmbH, Germany. Matrigel was kindly provided by Dr. Sanja Mijatovic from the Institute for Biological Research “Sinisa Stankovic”, University of Belgrade, Serbia. Propidium iodide (PI) was purchased from Roche Applied Science, Basel, Switzerland and Annexin-V-FITC (AV) from Abcam, Cambridge, UK. FITC-conjugated anti-P-gp antibody was provided by BD Biosciences, United Kingdom, while PE-conjugated anti-VEGF antibody was obtained from R&D Systems, Minneapolis, MN USA. Carboxyfluorescein succinimidyl ester (CFSE), dihydroethidium (DHE) was obtained from Molecular Probes®, Invitrogen, CA, USA. Primary antibodies against caspase 3 and β-actin were purchased from Cell Signaling Technology Inc, Danvers, MA, USA, while primary antibody against gamma-glutamylcysteine synthetase (γGCS) was a kind gift from Dr Bato Korac, Institute for Biological Research “Sinisa Stankovic”, University of Belgrade, Serbia. Peroxidase-conjugated goat anti-rabbit IgG was obtained from Jackson ImmunoResearch Laboratories Inc, West Grove, PA, USA.

### Cells and Cell Culture

NCI-H460 and U87 cell lines were purchased from the American Type Culture Collection, Rockville, MD. NCI-H460 cells were maintained in RPMI 1640 supplemented with 10% FBS, 2 mM L-glutamine, 4.5 g/L glucose, 10,000 U/mL penicillin, 10 mg/mL streptomycin, 25 µg/mL amphotericin B solution at 37°C in a humidified 5% CO_2_ atmosphere. NCI-H460/R cells were originally selected from NCI-H460 cells in our laboratory and cultured in a medium containing 100 nM DOX as described previously [9]. U87 cells were cultured in MEM supplemented with 10% FBS, L-glutamine (2 mM) and 5000 U/ml penicilin, 5 mg/ml streptomycin solution. U87-TxR cells were selected from U87 cells in our laboratory after continuous exposure to stepwise increasing concentrations of paclitaxel (100–300 nM) for a period of 9 months as already published [10]. HaCaT cell line (normal human keratinocytes obtained from CLS - Cell Lines Service, Eppelheim, Germany) was generous gift from Prof. Andra Jorg, Division of Biophysics, Research Center Borstel, Leibniz-Center for Medicine and Biosciences, Borstel, Germany. HaCaT cells were cultured in DMEM supplemented with 10% FBS, 4 g/L glucose, L-glutamine (2 mM) and 5000 U/ml penicilin, 5 mg/ml streptomycin solution. MDR cancer cells were sub-cultured at 72 h intervals using 0.25% trypsin/EDTA and seeded into a fresh medium at the following densities: 8,000 cells/cm^2^ for NCI-H460, 16,000 cells/cm^2^ for NCI-H460/R and U87, 32,000 cells/cm^2^ for U87-TxR. HaCaT cells were sub-cultured at 144 h intervals using 0.25% trypsin/EDTA and seeded into a fresh medium at 64, 000 cells/cm^2^.

### Sulforhodamine B Assay

Cells grown in 25 cm^2^ tissue flasks were trypsinized, seeded into flat-bottomed 96-well tissue culture plates and incubated overnight. Investigated cell lines NCI-H460, NCI-H460/R, U87, U87-TxR and HaCaT cells were seeded at 4, 000, 8,000, 8,000 and 16,000 cells/well, respectively. SF treatment (1–100 µM) lasted 72 h. The cellular proteins were stained with sulforhodamine B (SRB) assay, following slightly modified protocol of Skehan et al [11]. Briefly, the cells in 96-well plates were fixed in 50% trichloroacetic acid (50 µL/well) for 1 h at 4°C, rinsed in tap water and stained with 0.4% (w/v) sulforhodamine B in 1% acetic acid (50 µL/well) for 30 min at room temperature. The cells were then rinsed three times in 1% acetic acid to remove the unbound stain. The protein-bound stain was extracted with 200 µL 10 mM Tris base (pH 10.5) per well. The optical density was read at 540 nm, with correction at 670 nm (LKB 5060-006 Micro Plate Reader, Vienna, Austria).

### Matrigel Growth

For three-dimensional (3-D) cultures, cells were plated at the same densities as for two (2-D) cultures onto reconstituted (pre-gelled) basement membrane (Matrigel; BD Biosciences, San Jose, CA, USA) in RPMI 1640 medium with 10% FBS. Cells were incubated for 72 h and photographed live by phase microscopy.

### Determination of Cell Proliferation (CFSE Staining)

The rate of cell proliferation was measured by flow-cytometric analysis of cells labeled with carboxyfluorescein succinimidyl ester or CFSE [12]. Briefly, detached cells (5×10^6^ cells/mL) were stained with 1 µM CFSE for 10 min in dark at 37°C, washed twice in fresh medium, seeded in six-well plates at 5×10^4^ per well, and then exposed to SF. After 72 h of cultivation, cells were trypsinized and washed twice in PBS. Finally, the cells were resuspended in PBS and analyzed by flow-cytometry. Green fluorescence emission was measured using a FACSCalibur flow-cytometer (Becton Dickinson, Oxford, United Kingdom) and analyzed using Cell-Quest software.

### Cell Death Detection

The percentages of apoptotic, necrotic and viable cells were determined by Annexin-V-FITC (AV) and propidium iodide (PI) labeling. NCI-H460/R and U87-TxR cells were plated and incubated overnight in 6-well plates at density of 80,000 and 160,000 cells/well, respectively. After 72 h of SF treatment, the attached and floating cells were collected by centrifugation. The cells pellet was re-suspended in 100 µL of binding buffer containing 10 mM HEPES/NaOH, 140 mM NaCl, 5 mM CaCl_2_ (pH 7.4), supplemented with 0.2 µg AV and 1 µg PI. After the incubation period (30 min at 37°C in dark), additional 400 µL of binding buffer was added and AV/PI staining was analyzed within 1 h by flow-cytometry. The fluorescence intensity (green FL1-H and red FL2-H) was measured on FACSClibur flow-cytometer (Becton Dickinson, Oxford, United Kingdom). In each sample, 10,000 cells were recorded (gated to exclude cell debris), and the percentages of viable (AV− PI−), early apoptotic (AV+ PI−), apoptotic and necrotic (AV+ PI+), and already dead (AV− PI+) cells were analyzed by CellQuest Pro data analysis software.

### Caspase Activation

Activation of caspases was measured by flow-cytometry after labelling the cells with a cell-permeable, FITC-conjugated pan-caspases inhibitor (ApoStat; R&D Systems, Minneapolis, MN) according to the manufacturer`s instructions. The increase in green fluorescence (FL1-H) as a measure of caspase activity within individual cells of the treated population was determined using FACSCalibur flow-cytometer (Becton Dickinson, Oxford, United Kingdom).

### Autophagy Assessment

The appearance of acidic autophagic vesicles was detected by flow-cytometry. After SF treatment cells were trypsinized, washed and incubated for 15 min at 37°C with 1 µM acridine orange. Acridine orange-stained cell nuclei are fluorescent green, while autophagic lysosomes are fluorescent orange-red. The increase in red vs. green (FL3/FL1) fluorescence ratio, reflecting the autophagy, was determined using a FACSCalibur flow-cytometer (Becton Dickinson, Oxford, United Kingdom) and Cell Quest Pro software.

### Western Blot

Cells grown in 100 mm Petri dishes at following densities: 400,000 cells per dish for NCI-H460/R and 750,000 per dish for U87-TxR were lysed after SF treatment with lysis buffer (30 mM Tris-HCl pH 8.9, 150 mM NaCl, 1% NP-40) containing 1 mM phenylmethylsulfonyl fluoride and protease inhibitor cocktail (Sigma-Aldrich Chemie GmbH, Germany). After 30 min on ice, samples were centrifuged at 14 000 g for 15 min at 4°C, and supernatants were collected. Equal amounts of protein from each sample was separated by SDS-PAGE on 6–15% gels and transferred to nitrocellulose membranes (Bio-Rad, Hercules, CA, USA). Following incubation with primary antibodies against caspase 3, β-actin and gamma glutamylcstein synthetase (γGCS) and peroxidase-conjugated goat anti-rabbit IgG as the secondary antibody, specific protein bands were visualized using Amersham ECL reagent (GE Healthcare Life Sciences, UK). The protein levels of γGCS were quantified by densitometry using ImageJ software and expressed relative to β-actin.

### DHE Staining

Flow-cytometric measurements of dihydroethidium (DHE)-fluorescence were used to measure ROS concentration in MDR cancer cells. Adherent cells were rinsed with PBS and harvested by trypsinization. Cells were incubated in PBS with 10% FBS and 10 µM DHE for 45 min. DHE-fluorescence was analyzed by flow-cytometry (excitation 488 nm, and emission 585 nm, FL2-H channel). Mean fluorescence intensity (MFI) was calculated after correction for autofluorescence.

### Colorimetric Detection of Glutathione (GSH)

Cells grown in 25 cm^2^ tissue flasks were trypsinized and counted. The same number of cells (2.5×10^6^) for each sample was exposed to further procedure. Briefly, the cells were collected by centrifugation at 700×g for 5 minutes at 4°C and the supernatant was removed. Then, the cell pellet was resuspended in 0.5 ml ice-cold PBS and centrifuged at 700×g for another 5 minutes at 4°C. The supernatant was removed and the cells were lysed in 80 µl ice-cold Glutathione Buffer (GSH Colorimetric Detection Kit, Bio-Vision, CA) for 10 minutes on ice. Then, 20 µl of 5% Sulfosalicylic Acid was added and the samples were centrifuged at 8000×g for 10 minutes at 4°C. The supernatant was transferred to a fresh tube and used for GSH assay. Glutathione Buffer was added to each well (96-well plate) at a volume of 160 µl and incubated 10 minutes at room temperature. Afterwards, 20 µl of either prepared standards or samples was added to each well and incubated for another 10 minutes at room temperature. Finally, 20 µl of Substrate Solution (GSH Colorimetric Detection Kit, BioVision, CA) was added and the absorbance of generated product (2-nitro-5-thiobenzoic acid) was read at 405 nm (LKB 5060–006 Micro Plate Reader, Vienna, Austria). The concentrations of GSH were determined by using the standard GSH calibration curve and related to the concentrations of proteins in cell lysates. The GSH detection for each sample was performed at least six times.

### RNA Extraction and RT-PCR

Total RNA was extracted from untreated NCI-H460/R and U87-TxR cells and the cells treated with SF. The isolation was carried out using Trizol (Invitrogen Life Technologies, CA, USA) according to the manufacturer’s instructions. RNA was quantified on spectrophotometer and quality was determined by agarose gel electrophoresis. Reverse transcription (RT) reactions using 25 µg total RNA were performed with oligo-dT primers using M-MLV Reverse Transcriptase (Gibco BRL, USA) following the manufacturer’s instructions. PCR reactions were performed with primers specific for, *gst-π*, *vegf*, *mdr1* and *hif-1α* [13]–[16], β*-actin* [17] and *gapdh* [18] was used as an internal control and co-amplified with genes of interest in all PCR experiments.

The PCR reactions were performed on the GeneAmp® PCR System 9700 (Applied Biosystems, CA, USA) under the following conditions for *hif-1α*, *mdr1* and *gst-π*: initial denaturation at 95°C for 5 min, 24, 25 or 28 cycles (respectively) at 95°C for 15 s, 56°C for 30 s, 72°C for 30 s and at 4°C indefinitely. When PCR was performed to determine the expression of the *vegf* gene, 35 cycles were applied with the annealing temperature of 62°C. The *gapdh* primers were used at following ratios: 1∶4 to the *mdr1* primers and 1∶6 to the *hif-1α* primers in order to attain linear amplification conditions. The β*-actin* primers were used at following ratios: 1∶2 to the *gst-π* primers and 1∶5 to the *vegf* primers in order to attain linear amplification conditions. The PCR products were separated in 2% agarose gels stained with ethidium bromide. Multi-Analyst/PC Software Image Analysis (Bio-Rad Gel Doc 1000, CA, USA) was employed for densitometry analysis.

### DOX Accumulation Assay

DOX accumulation was analyzed by flow-cytometry utilizing the ability of DOX to emit fluorescence. The intensity of the fluorescence was proportional to DOX accumulation. Studies were carried out after 24 h, 48 h and 72 h SF treatment. NCI-H460/R and U87-TxR cells were cultured in 25 cm^2^ flasks, trypsinized and re-suspended in 10 mL centrifuge tubes in a DOX-containing medium (20 µM). Then, the cells were incubated at 37°C in 5% CO_2_ for 120 min. At the end of the accumulation period, the cells were pelleted by centrifugation, washed with phosphate buffered saline (PBS) and placed in cold PBS. The samples were kept on ice in dark until the analysis on FACScalibur flow-cytometer (Becton Dickinson, Oxford, United Kingdom). The fluorescence of DOX was assessed on fluorescence channel 2 (FL2-H) at 530 nm. A minimum of 10,000 events were assayed for each sample. The differences in curve shape were quantified using a Komogorov-Smirnov nonparametric statistic. P values were calculated (available on request) in CellQuest Pro and run on a Macintosh computer.

### Flow-cytometric Analysis of P-gp and VEGF Expression

Flow-cytometry was used to measure P-gp and VEGF expression levels in MDR cancer cells. Untreated and SF treated cells (2×10^5^) were collected by trypsinization, washed in ice-cold PBS, and then directly immuno-stained by FITC-conjugated anti-P-gp antibody according to the manufacturers’ protocol (BD Biosciences, United Kingdom). An isotype control IgG2bκ (Abcam, Cambridge, United Kingdom) was evaluated for each experimental sample to discriminate the level of background fluorescence of negative cells. For VEGF expression analysis, the cells were fixed in 4% paraformaldehyde, 10 min at room temperature, washed and resuspended at saponin 0.05% (w/v) buffer and incubated with PE-conjugated anti-VEGF antibody according to the manufacturers’ protocol (R&D Systems, USA). An isotype control IgG2a (Abcam, Cambridge, United Kingdom) was evaluated for each experimental sample to discriminate the level of background fluorescence of negative cells. Mean fluorescence intensity was determined for positively stained cells. The samples were kept on ice in dark until the analysis on FACScalibur flow-cytometer (Becton Dickinson, Oxford, United Kingdom). The fluorescence of FITC-conjugated anti-P-gp was assessed on fluorescence channel 1 (FL1-H) at 530 nm, while PE-conjugated anti-VEGF was assessed on fluorescence channel 2 (FL2-H) at 585 nm. A minimum of 10,000 events were assayed for each sample (the gate excluded cell debris and dead cells) and the obtained results were analysed using Cell Quest Pro Software (Becton Dickinson, Oxford, United Kingdom).

### MTT Assay

Cell metabolic activity was assessed by the MTT assay based on the reduction of 3-(4,5-dimethyl-2-thizolyl)-2,5-diphenyl-2H-tetrazolium bromide (MTT, Sigma, St Louis, MO) into formazan dye by active mitochondria of living cells. The combined effects of simultaneous and subsequent treatment were studied on MDR cancer cell lines. NCI-H460/R and U87-TxR cells prepared for simultaneous treatment were seeded at 4, 000 and 8,000 cells/well, respectively. SF treatment (5 µM) in combination with different DOX concentrations lasted 72 h. The subsequent treatments were performed on NCI-H460/R and U87-TxR cells initially seeded at lower densities (500 cells/well and 1,000 cells/well, respectively). Pretreatment with 5 µM SF lasted for 72 h and was followed by additional 72 h treatment with different concentrations of DOX. MTT was added to final concentration of 0.1 mg/ml in each well of a 96-well microplate and plates were incubated at 37°C for 4 h. Then, DMSO was added to dissolve formazan product, which amount was proportional to the number of live cells. The absorbance of dissolved dye was measured at 540 nm using an automatic microplate reader (LKB 5060-006 Micro Plate Reader, Vienna, Austria). Growth inhibition (I) was determined according to the following equitation:
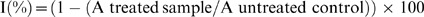
where A is for absorbance.

IC_50_ value was defined as concentration of each drug that inhibited cell growth by 50%. IC_50_ was calculated by linear regression analysis using Excel software.

### ELISA for Detection of Human VEGF_165_ in Cell Culture Medium

MDR cells (NCI-H460/R and U87-TxR), seeded in 6-well plates, were incubated overnight and then treated with SF. The cell medium (supernatant) was collected 24 h, 48 h and 72 h after treatment for determination of secreted VEGF_165_ protein by VEGF immunoassay kit (Quantikine Human VEGF ELISA Kit, R&D Systems, Minneapolis, USA). The procedure was complied according to the manufacturers’ manual. The results were normalized based on the same amount of cells analyzed. A standard curve was generated using recombinant VEGF_165_ supplied with the kit. The concentrations of VEGF in cell-free culture supernatants were examined in triplicates in two independent experiments.

### Statistical Analysis

Statistical analysis was performed by Statistica 6.0 software. The results were tested for normality. If obtained values were not normally distributed, the groups were compared by Student’s *t* – test. For normally distributed variables, one-way analysis of variance (ANOVA) was used. When statistical significance was observed, the Tukey honest significant difference (HSD) test was applied. Statistical significance was accepted if p<0.05 (*), p<0.01 (**), p<0.001 (***).

## Results and Discussion

### SF Inhibits the Growth of MDR Cancer Cells

We established NSCLC and glioblastoma P-gp over-expressing cell lines (NCI-H460/R and U87-TxR) with MDR phenotype in order to investigate potential anti-cancer agents [9], [10]. NCI-H460/R and U87-TxR are MDR cancer cell lines that originated from NCI-H460 (NSCLC cell line) and U87 (glioblastoma cell line). The parental cell lines were considered as sensitive since the cells derived from patients who had not undergone chemotherapy. In the present study, we tried to elucidate the action of sulfinosine (SF), a synthetic purine nucleoside analog, in these two MDR cancer cell lines. We choose SF because of evidences that its therapeutically effective concentrations could not induce the resistance. SF also efficiently penetrates to CNS [2]. Moreover, recent clinical study showed that the combination therapy including 6-thioguanine (closely related molecule to SF) is promising for patients with recurrent high-grade anaplastic glioma [19].

**Figure 2:**
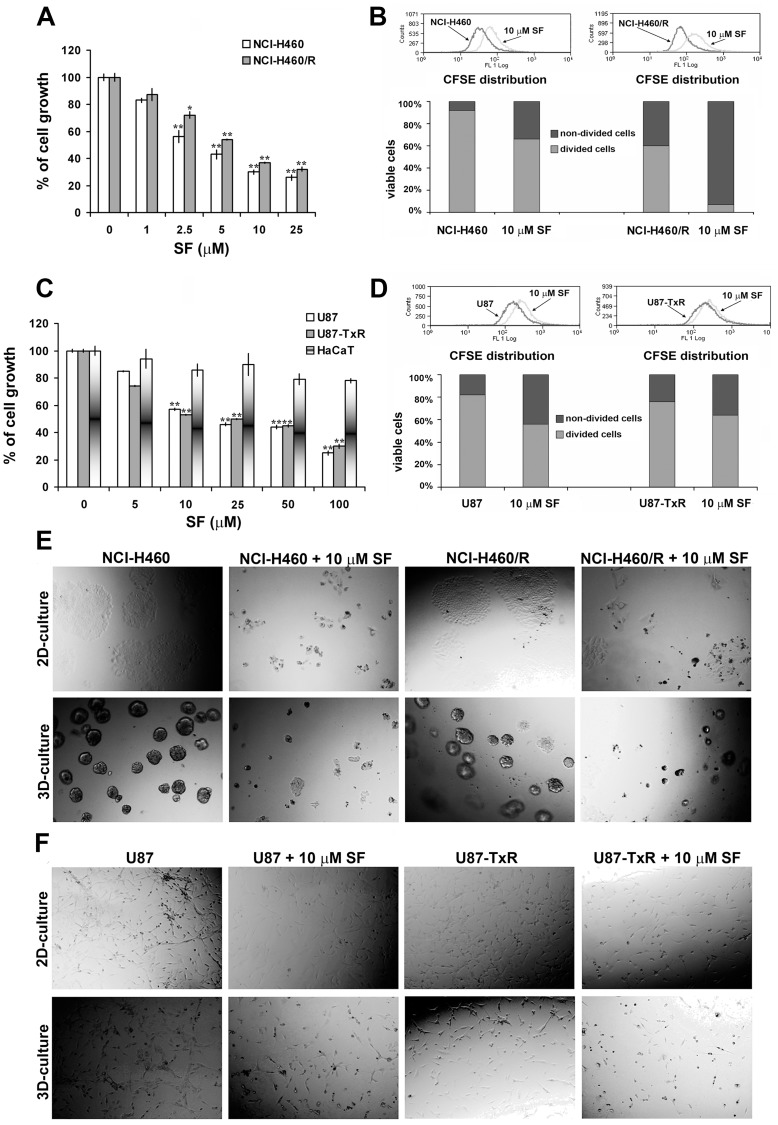
SF inhibits the cell growth and suppresses the cell proliferation. The growth inhibitory effects of SF on NCI-H460 and NCI-H460/R (**A**), U87, U87-TxR and HaCaT (**C**) cells grown on plastic after 72 h treatment were assessed by SRB assay. Average ± S.D. values were calculated from five independent experiments (n = 5). NCI-H460 and NCI-H460/R (**B**), U87 and U87-TxR (**D**) cells were stained with CFSE and incubated for 72 h with 10 µM SF. The rate of proliferation (CFSE declination) was determined by flow-cytometry on channel FL1. Light microscopy of NCI-H460 and NCI-H460/R (**E**), U87 and U87-TxR (**F**) cell growth on plastic (2-D culture) and matrigel growth (3-D culture) after 72 h of 10 µM SF treatment.

The effects of SF on cancer cell growth after 72 h treatment were evaluated by the chemo-sensitivity assay - sulforhodamine B (SRB). SF inhibited the growth of sensitive and MDR cancer cell lines in a dose-dependent manner (Figure 2A, C). Since application of anti-cancer agents is limited by their toxicity towards normal cells, we tested the effect of SF on HaCaT cells (normal human keratinocytes). The effects on growth of HaCaT after continuous treatment of 72 h were assessed also by SRB assay. SF did not reduce significantly the number of normal cells even with the highest concentration (100 µM) (Figure 2C). We demonstrated that SF inhibits the growth of sensitive as well as resistant NSCLC and glioblastoma cells in micro-molar range of concentrations, and that its efficacy was not affected by the presence of the MDR phenotype. Moreover, SF was non-toxic to normal cells (HaCaT) in the range of concentrations necessary to inhibit the growth of cancer cells.

Next, we evaluated the cytostatic effect of 10 µM SF in each cell line by CFSE staining (Figure 2B, D). CFSE is a vital dye stable in the cytoplasm for about 7–8 generations, but the intensity of CFSE fluorescence declines due to its progressive halving within daughter cells following each cell division. In that way, the CFSE distribution in the cells can estimate the rate of cell proliferation. The CFSE distribution in control and SF treated samples is illustrated by flow-cytometric profiled histograms (Figure 2B, D). The inhibition of proliferation observed in the presence of SF was the most pronunced in NCI-H460/R cells. These results indicated that the inhibition of proliferation is partly responsible for the anti-cancer activity of SF.

Since NSCLC and glioblastoma cell lines have high metastatic potential, we compared the effects of SF to inhibit the cell growth after 72 h on plastic (2-D culture) and in reconstituted basement membrane – matrigel (3-D culture) (Figure 2E, F). We found that SF inhibited the growth in 3-D culture with the same efficacy observed in 2-D culture. The fact that the cells were detached from each other after SF treatment in matrigel, especially glioblastoma cells, points to the change in their adhesive properties. Therefore, we speculate that SF could affect the affinity of cancer cells to invade the blood vessels, induce tumor-angiogenesis and metastasis.

### SF Induces Caspase-dependent Apoptosis in MDR Cancer Cells

**Figure 3:**
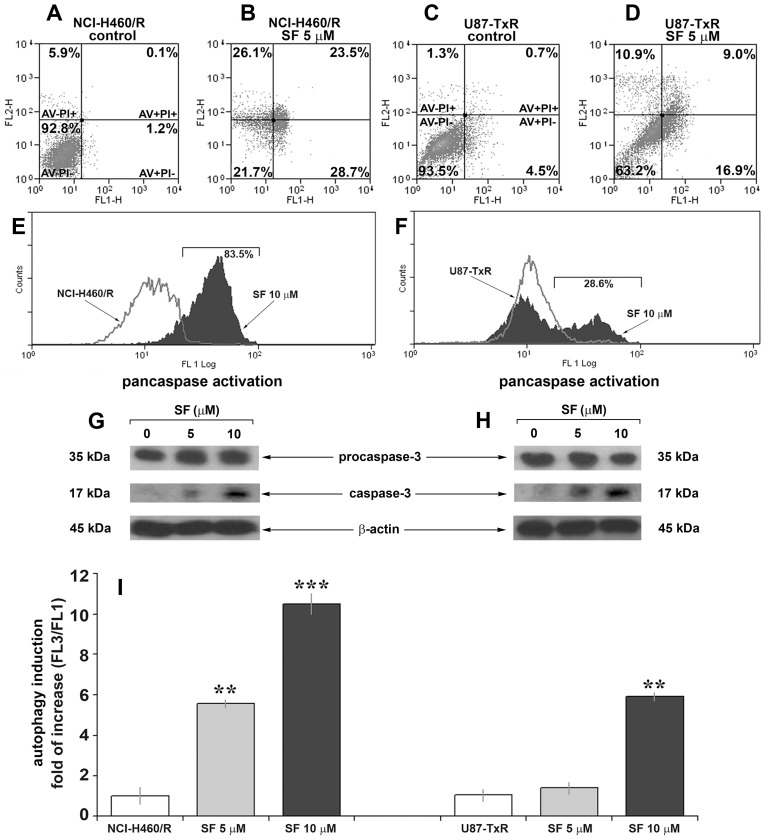
SF induces caspase activation, apoptosis and autophagy in MDR cancer cell lines. Cell death analysis of NCI-H460/R (**A**) and U87-TxR (**C**) cells untreated and treated with 5 µM SF (**B**, **D**) for 72 h. The samples were analyzed for green fuorescence (Annexin-V-FITC) and red fluorescence (Propidium Iodide) by flow-cytometry. The assay distinguishes viable cells (AV− PI−), apoptotic cells (AV+ PI−), late apoptotic and necrotic cells (AV+ PI+) and secondary necrotic or dead cells (AV− PI+). Pancaspase activation in NCI-H460/R (**E**) and U87-TxR (**F**) cells untreated and treated with 10 µM SF was assessed after 72 h by flow-cytometry. The cells were labelled with a cell-permeable, FITC-conjugated pan-caspases inhibitor (ApoStat). Western blot analysis of procaspase-3, p17 cleaved caspase-3 and β-actin in NCI-H460/R (**G**) and U87-TxR (**H**) cells. (**I**) The autophagy in NCI-H460/R and U87-TxR cell lines assessed after 144 h incubation period (72 h of SF treatment followed by next 72 h of cell recovery in fresh medium). Fold of increase in red vs. green (FL3/FL1) fluorescence ratio after acridine orange-staining was determined using a FacsCalibur flow cytometer and Cell Quest Pro software. The statistical significance between the controls and treatments is presented as p<0.01 (**) and p<0.001 (***).

Next, we proceeded to examine whether induction of apoptosis contributes to the anti-cancer action of SF in MDR cancer cell lines. To assess the apoptosis induced by SF after 72 h the cells were seeded at optimal density for their growing properties. In that way, the untreated controls did not achieve confluence at the end of incubation period. Annexin-V-FITC/Propidium Iodide staining revealed that SF increases the proportion of apoptotic cells (AV+PI−) in both MDR cancer cell lines. The results are summarized in (Figure 3A, B, C, D): alive cells are negative for both, Annexin-V and Propidium Iodide (AV−PI−); apoptotic cells only bind Annexin-V (AV+ PI−), apoptotic and necrotic cells are Annexin-V and Propidium Iodide positive (AV+ PI+) and secondary necrotic cells, that were already dead, are positive only for Propidium Iodide (AV− PI+). The percentages of apoptotic and necrotic cells were increased in samples treated with 5 µM SF (Figure 3B, D).

Caspases play a key role in the apoptotic pathway. Initiator caspases, which include caspases-2, -8, -9 and -10, activate the caspase cascade through the removal of the inactive prodomains of the effector caspases. Once activated, effector caspases, including caspases-3, -6 and -7, cleave several dozen key substrates within the cell in order to carry out the apoptotic process [20]. We showed by a fluorochrome-labeled pan-caspase inhibitor ApoStat that SF induced the activation of caspases (Figure 3E, F). SF caused the shift of ApoStat flow-cytometric profile by 83.5% and 28.6% in NCI-H460/R and U87-TxR cells, respectively (Figure 3E, F). We found that the induction of apoptosis by SF after 72 h was caspase-dependent in both resistant cell lines. However, the caspase activation was more pronounced in resistant NSCLC than in glioblastoma cells. In order to find if an effector caspase-3 was activated after SF treatment, we analyzed the expression of its precursor procaspase-3 (35 kDa) and cleaved caspase-3 (17 kDa) by Western blotting (Figure 3G, H). The results revealed the obvious increase in 17 kDa form after 5 and 10 µM SF application in both MDR cancer cell lines. Although, the expression of procaspase-3 was not affected by SF treatment, the increase of cleaved form corresponds to the activation of caspase-3.

Our previous study revealed that *p53* has undergone mutations during induction of resistance in NSCLC (NCI-H460/R) cells [6]. *p53* mutation is often associated with increased resistance to chemotherapy [21]. However, the anti-cancer action of SF certainty included induction of apoptosis in resistant NSCLC cells. The proposed mechanism for SF anti-cancer action involves the formation of adducts with sulfhydryl compounds - glutathione and cysteine [22], [23]. Depletion of glutathione pool may cause down-regulation of Bcl-2 [24], release of cytochrom *c* from mitochondria and activation of caspases [25]. Therefore, the cell death induced by SF in resistant NSCLC cell line could be p53-independent.

Several anti-apoptotic mechanisms, such as over-expression of PKA, HSP70, Bcl-2 and deficiency in PTEN are responsible for the evading apoptosis in glioblastoma sensitive cell line (U87) [26]–[29]. We assume that the same mechanisms are present in resistant glioblastoma cell line (U87-TxR). However, U87-TxR cells were susceptible to the induction of cell death by SF.

Autophagy, a catabolic process responsible for the removal of long-lived proteins and damaged organelles through the lysosomal machinery, and apoptosis may be induced by common upstream signals, and thus results in combined autophagy and apoptosis. Under certain circumstances, autophagy constitutes a stress adaptation that avoids cell death and suppresses apoptosis [30]. In order to reveal the possible connection between SF driven apoptosis and autophagy, we investigated by flow-cytometry whether SF could induce the appearance of autophagy-associated acidic vesicles (Figure 3I). The changes in the level of autophagy were obtained after removal of SF from medium. Significant increases in autophagy were observed after 5 and 10 µM SF treatments in NCI-H460/R and U87-TxR cells, respectively (Figure 3I).

### SF Modulates Detoxification Capacity of MDR Cancer Cells

**Figure 4:**
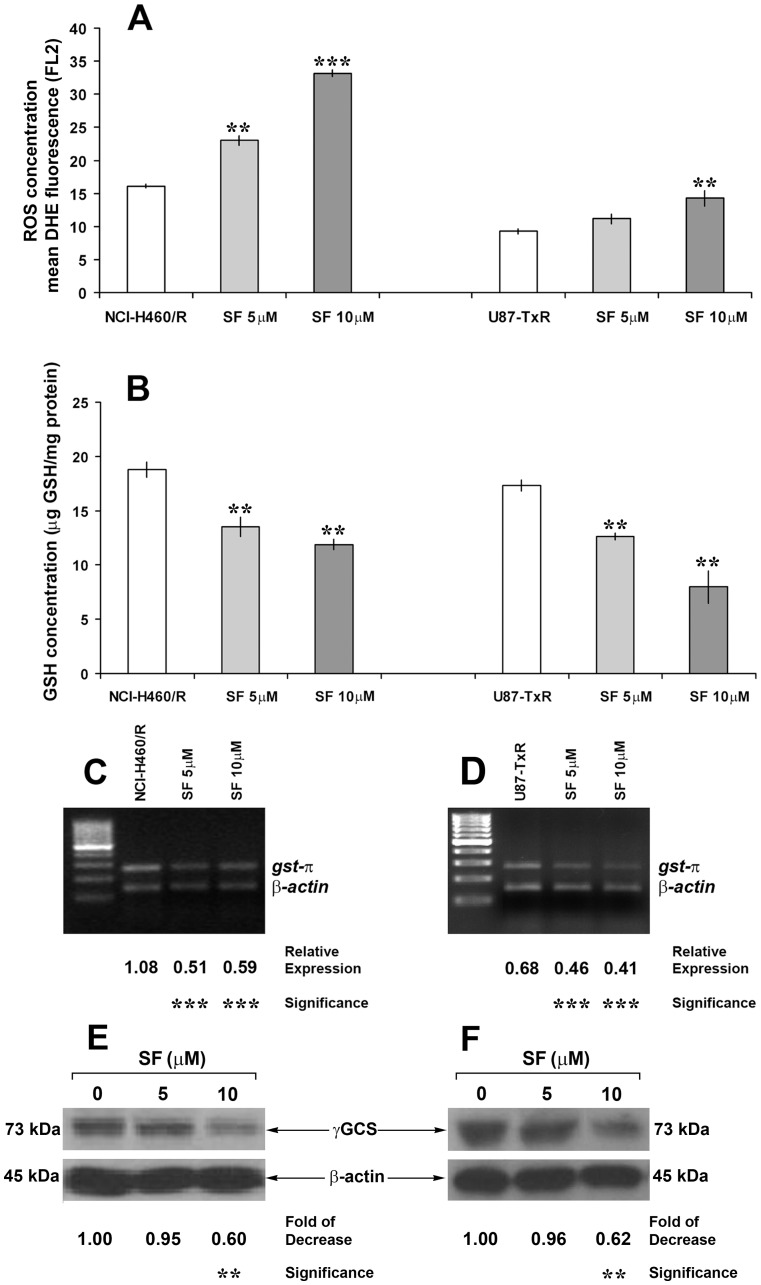
SF increases ROS concentration and inhibits GSH detoxification in MDR cancer cell lines. Flow-cytometric measurements of dihydroethidium (DHE)-fluorescence correspond to cellular ROS levels (**A**). Mean fluorescence intensity (MFI) calculated after correction for autofluorescence is presented. The data indicate the average ± S.D. of three experiments carried out in triplicate. Statistical significance of SF treatment compared to untreated control: p<0.01 (**) and p<0.001 (***). Detection of GSH concentration (**B**) for each sample was performed at least six times. The data indicate the average ± S.D. Statistical significance of SF treatment compared to untreated control: p<0.01 (**). The expression of *gst-π* mRNA in NCI-H460/R (**C**) and U87-TxR cells (**D**) was evaluated relative to the internal control - *β-actin*. The PCR products were separated on agarose gels beside a 100 bp DNA ladder. Statistical significance between treated and untreated cells is presented as p<0.001 (***).The decreases in γGCS expression in NCI-H460/R (**E**) and U87-TxR cells (**F**) after SF treatments were calculated relative to β-actin expression and untreated samples. Statistical significance between treated and untreated cells is presented as p<0.01 (**).

Cellular redox environment is a delicate balance between the levels of reactive oxygen species (ROS i.e., superoxide and hydrogen peroxide) and the antioxidant system that scavenges them (i.e., glutathione/glutathione peroxidase and tioredoxin/peroxiredoxin pathways) [31]. Considering that ROS can be generated from exogenous sources (e.g. ionizing radiation, chemicals), we examined whether SF changes the ROS concentration in MDR cancer cells. We used dihydroethidium (DHE)-fluorescence and flow-cytometry to measure the ROS levels. DHE is a non-fluorescent chemical that upon entry into the cell undergoes oxidation to yield the fluorescent chemical, 2-hydroxyethidium (HE). HE intercalates with DNA and shows strong fluorescence in presence of superoxide radical [32]. Indeed, 10 µM SF significantly increased the ROS concentration in both resistant cancer cell lines, while even 5 µM SF induced considerable change in NCI-H460/R cells (Figure 4A).

The main cellular antioxidant detoxification pathway – GSH system scavenges ROS and prevents them from causing intracellular damage including lipid peroxidation, DNA damage and protein oxidation [33]. Therefore, we studied the effects of SF on GSH concentration. The concentration of reduced glutathione (GSH) was measured in untreated cells and cells treated for 72 h with 5 and 10 µM SF. SF caused strong and significant depletion of GSH in both resistant cancer cell lines (Figure 4B). After 10 µM SF treatments, the concentrations decreased from19 to 12 µg GSH/ml protein in NCI-H460/R and from 17 to 7 µg GSH/ml protein in U87-TxR cells (Figure 4B).

We also measured the level of mRNA expression of the common drug resistance marker glutathione-S-transferase π (*gst-π*), which is a component of GSH system. Application of SF to the resistant cell lines induced a significant reduction in *gst-π* expression compared to untreated cells (Figure 4C, D). Herein, we also showed that 10 µM SF significantly decreased the expression of gamma Glutamyl-cysteine-synthetase (γGCS), the enzyme critical for GSH synthesis (Figure 4E, F).

The role of GSH and related enzymes in cellular resistance to xenobiotics, including chemotherapy, is well established. Clearly, SF has a potential to modulate GSH system to therapeutic advantage. SF significantly decreased intracellular GSH levels by inhibition of its synthesis. This contributes further to the increase of ROS, the induction of cell damage and eventually the cell death.

### SF Decreases the Expression of *hif-1α* and *hif-1α* Regulated mRNAs

**Figure 5:**
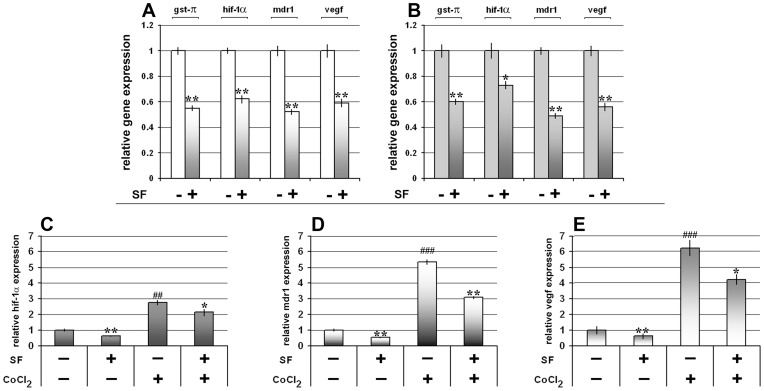
SF decreases the expression of mRNAs involved in tumor progression. The expression of *gst-π*, *hif-1α*, *mdr1* and *vegf* mRNAs in NCI-H460/R (**A**) and U87-TxR cells (**B**) after 10 µM SF treatment was evaluated relative to the internal control – *gapdh* and *β-actin*, and calculated in relation to the untreated control. Statistical significance between treated and untreated cells is presented as p<0.05 (*) and p<0.01 (**). Relative *hif-1α* (**C**), *mdr1* (**D**) and *vegf* (**E**) mRNAs expression after 10 µM SF treatment in normoxic (absence of CoCl_2_) and hypoxic (presence of 50 µM CoCl_2_) conditions studied in NCI-H460/R cells. Statistical significance between SF treated and SF untreated cells is presented as p<0.05 (*) and p<0.01 (**).Statistical significance between CoCl_2_ treated and CoCl_2_ untreated cells is presented as p<0.01 (##) and p<0.001 (###).

We assumed that SF action through modification of redox status (GSH depletion and ROS generation) could influence the expression level of hypoxia inducible factor-1α (HIF-1α) and indirectly decrease the expression of P-gp and VEGF. We found that 10 µM SF significantly decreased the mRNA battery (*gst-π*, *hif-1α*, *mdr1* and *vegf*) in both MDR cancer cell lines (Figure 5A, B). In order to reveal whether SF could regulate the HIF-1α expression induced by hypoxia, MDR cancer cells were treated with cobalt chloride (CoCl_2_) that stabilizes HIF-1α and induces HIF-1 responsive genes similarly to that of hypoxic condition [16]. We showed that SF retained the potential to inhibit the expression of *hif-1α*, *mdr1* and *vegf* mRNAs even in hypoxic mimic condition in NCI-H460/R cell line (Figure 5C, D, E).

### SF Inhibits P-gp Expression and Activity

**Figure 6:**
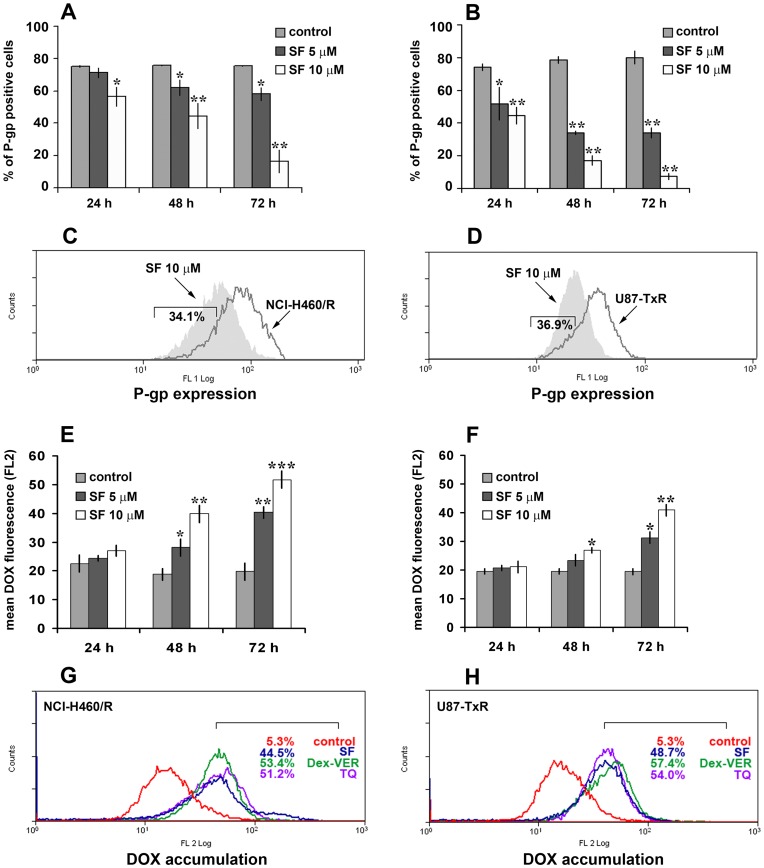
SF inhibits P-gp expression and increases DOX accumulation. P-gp expression was detected after 24 h, 48 h and 72 h in NCI-H460/R (**A**) and U87-TxR (**B**) cells untreated and treated with 5 and 10 µM SF. The percentage of P-gp positive cells (stained with FITC-labeled direct antibody) was determined after elimination of the cells that were stained with FITC-labeled isotypic antibody. The corresponding flow-cytometric histograms illustrate the effect of 10 µM SF after 72 h in NCI-H460/R (**C**) and U87-TxR (**D**) cells. DOX accumulation in NCI-H460/R (**E**) and U87-TxR (**F**) cells untreated and pretreated with, 5 and 10 µM SF. DOX accumulation was assessed at each time point (24 h, 48 h and 72 h) after 120 min of 20 µM DOX treatment. Flow cytometric profiles after 72 h pretratment compare the effects of 10 µM SF, 50 nM TQ and 10 µM Dex-VER in NCI-H460/R (**G**) and U87-TxR (**H**) cells Three independent experiments were performed (a minimum of 10,000 events were collected for each experimental sample). Statistical significance between treated and untreated cells is presented as p<0.05 (*), p<0.01 (**) and p<0.001 (***).

Considering the obtained results on mRNAs expression, especially *mdr1* gene, next, we studied the potential of SF to modulate MDR. Therefore, we assessed the P-gp (coded by *mdr1*) expression by flow-cytometry (Figure 6A, B, C, D). We used direct FITC-labeled antibody for P-gp to study the changes in protein expression level. After determination of background fluorescence with isotypic control antibody, we were able to define the portion of P-gp positive cells in each tested sample. Significant decreases in amount of P-gp positive NCI-H460/R and U87-TxR cells were observed after SF treatment (Figure 6A, B). The effect of SF on P-gp expression was concentration- and time-dependent. The best effect was achieved after 72 h and illustrated by flow-cytometric profiled histogram (Figure 6C, D). The fluorescence in SF treated samples declined in comparison to untreated controls of NCI-H460/R and U87-TxR by 34.1% and 36.9%, respectively. SF clearly decreased the fraction of P-gp expressing cells in both resistant cancer cell lines.

In order to investigate the effect of SF on P-gp function in NCI-H460/R and U87-TxR cell lines, we analyzed intracellular accumulation of chemotherapeutic (DOX), which is P-gp substrate (Figure 6E, F, G, H). We confirmed that MDR in NCI-H460/R and U87-TxR cell lines correlates with over-expression of P-gp membrane transporter. Low intracellular accumulation of P-gp substrates – DOX and Rhodamine 123 shown in these cell lines was the consequence of high P-gp activity [9], [10].

The accumulation of DOX was assessed by flow-cytometry and compared among SF, TQ (non-competitive P-gp inhibitor) and Dex-VER (competitive P-gp inhibitor). Marked increases in DOX accumulation were observed in NCI-H460/R and U87-TxR cells pretreated with SF during 48 h and 72 h (Figure 6E, F). SF was the most efficient in the modulation of P-gp activity after 72 h showing the same potential as TQ and Dex-VER in both MDR cancer cell lines (Figure 6G, H). We verified that the effect of SF on DOX accumulation was concentration- and time-dependent. The effect on P-gp expression was in accordance with obtained increases in DOX accumulation. We showed that the decrease in P-gp expression correlates with the strongest inhibition of P-gp activity after 72 h of SF application. Therefore, we confirmed the P-gp modifying effect of SF in two different MDR cancer cell lines.

### SF Chemo-sensitizes MDR Cancer Cells

Since SF significantly inhibits P-gp activity and expression, we decided to combine SF and DOX with the expectation that SF would improve DOX anti-cancer action. The effects of 5 µM SF on the chemo-sensitivity of MDR cancer cell lines were assessed by the MTT assay. We chose 5 µM SF as relevant concentration, which considerably decreased the percentage of P-gp positive cells in both MDR cancer cell lines. In order to distinguish the possible synergistic interaction of DOX and SF from chemo-sensitizing effect of SF, we compared the effects of simultaneous and subsequent treatment (Table 1). The IC50 value for DOX decreased in NCI-H460/R cells from 3.250 µM to only 3.036 µM in simultaneous treatment with SF. However, SF promoted significant reversal of resistance to DOX (7.26-fold) in subsequent treatment by decreasing the IC50 value for DOX from 7.083 µM to 0.976 µM (Table 1). SF also achieved significant chemo-sensitizing effect in U87-TxR cells in subsequent treatment promoting 17.01-fold reversal of DOX resistance (Table 1). The decrease in IC50 value for DOX in simultaneous treatment with SF (from 0.224 µM to 0.098 µM) was not significant in U87-TxR cells. We confirmed the chemo-sensitizing effect of SF in subsequent treatment with DOX. However, the improvement of DOX effect in simultaneous treatment with SF was negligible. In previous study, we showed that SF decreased the expression of topo IIα, which is the main target of DOX [5]. It implies that the effect of these two drugs in combination should be antagonistic. Therefore, the synergy between SF and DOX observed in subsequent treatment is obviously the result of SF facilitated DOX accumulation and the chemo-sensitization achieved by SF could not be merely the consequence of SF and DOX intracellular interaction.

**Table 1:** Relative reversion of resistance to DOX in simultaneous and subsequent treatments with SF.

Cell Lines	Drugs	IC_50_ (µM)	Relative Reversion
**NCI-H460/R**	DOX	3.250±0.260	
	SF 5 µM (+ DOX)		
	simultaneously	3.036±0.091	1.07
	DOX	7.083±0.212	
	SF 5 µM (+ DOX)		
	subsequently	0.976±0.010	**7.26**
**U87-TxR**	DOX	0.224±0.027	
	SF 5 µM (+ DOX)		
	simultaneously	0.098±0.025	2.28
	DOX	4.100±0.079	
	SF 5 µM (+ DOX)		
	subsequently	0.241±0.019	**17.01**

In this manner, we showed that SF significantly enhanced DOX cytotoxicity in subsequent treatment. Our results suggest that the improvement of DOX cytotoxicity was caused by SF induced inhibition of P-gp expression and activity. In favor to this are also the evidences that purine analogs could alter membrane glycoprotein synthesis [34].

### SF Modulates the Intracellular Pool of VEGF_165_ in MDR Cells

Vascular endothelial growth factor (VEGF) mediates pro-angiogenic effects. VEGF expression has been found to significantly correlate with new vessel formation and tumor progression in patients with NSCLC [35], while gliomas are reputed for their high micro-vascular proliferation [36]. New targeting strategies are aimed to block neo-angiogenesis in these two malignancies [37], [38].

Since it was shown that 6-thioguanine has anti-angiogenic potential [4], we decided to examine the effect of SF on gene expression and protein secretion of VEGF_165_ in resistant NCI-H460/R and U87-TxR cells. Additionally, VEGF and P-gp expression are regulated by the same transcriptional factor – hypoxia inducible factor-1α (HIF-1α) [39], and high concentrations of VEGF could decrease the expression of P-gp [40], [41].

**Figure 7:**
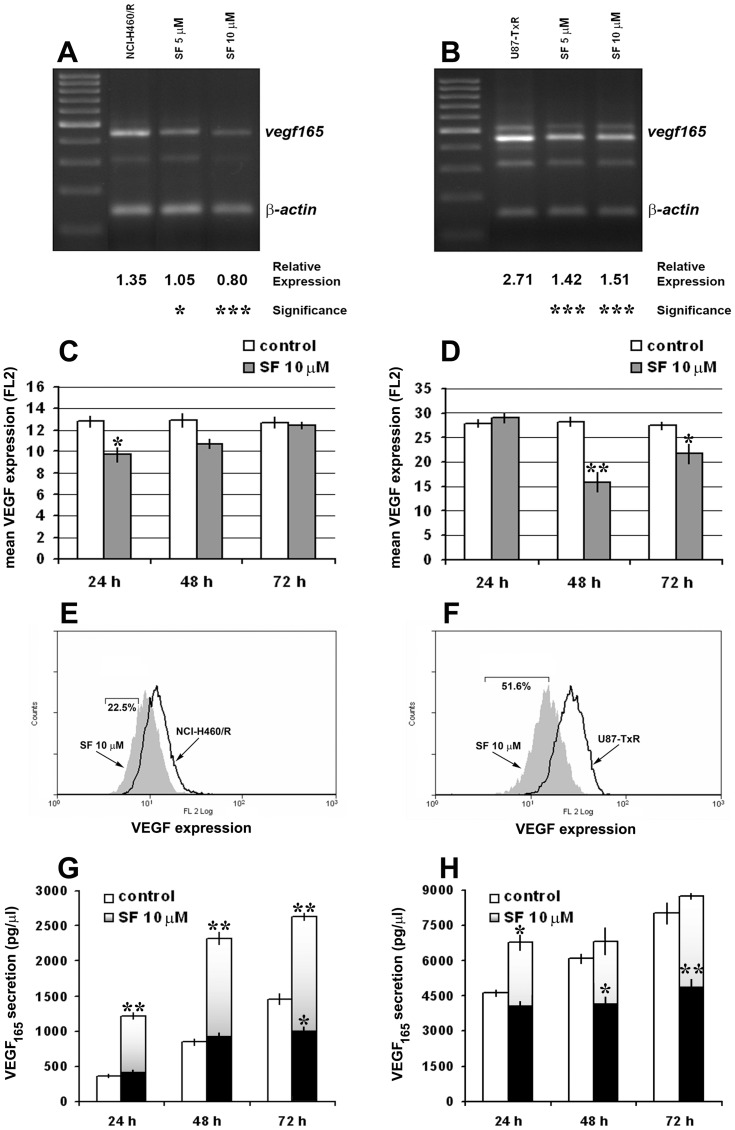
SF modulates VEGF_165_ expression and secretion in MDR cancer cell lines. The amplified NCI-H460/R (**A**) and U87-TxR samples (**B**) of the *vegf* gene (four splicing products of *vegf* mRNA) were visualized with ethidium-bromide in agarose gel next to a DNA ladder (100 bp). The PCR product of *β-actin* was co-amplified with *vegf*. The relative expression of *vegf165* was calculated in relation to *β-actin* expression. Statistical significance between treated and untreated cells is presented as p<0.05 (*) and p<0.001 (***). VEGF expression was detected after 24 h, 48 h and 72 h in NCI-H460/R (**C**) and U87-TxR (**D**) cells untreated and treated with 10 µM SF. The mean fluorescence of VEGF (PE-labeled direct antibody) was determined after elimination of the signal obtained with PE-labeled isotypic antibody. The corresponding flow-cytometric histograms illustrate the effect of 10 µM SF after 24 h in NCI-H460/R (**E**) and 48 h in U87-TxR (**F**) cells. VEGF secretion levels in culture medium were evaluated after 24 h, 48 h, and 72 h using Human VEGF Immunoassay Kit. NCI-H460/R (**G**) and U87-TxR cells (**H**) were treated with 10 µM SF. The data indicate the average ± S.D. of four experiments carried out in triplicate. Statistical significance of SF treatment compared to untreated control when the secretion levels were normalized based on the same amount of cells analyzed in untreated and treated samples, grey bars: p<0.05 (*) and p<0.01 (**). Statistical significance of SF treatment compared to untreated control when the secretion levels of treated cells were not normalized, black bars: p<0.05 (*) and p<0.01 (**).

We examined the changes in *vegf165* mRNA expression levels in NCI-H460/R (Figure 7A) and U87-TxR cells (Figure 7B) treated with 5 and 10 µM. The reduction of *vegf165* mRNA expression in NCI-H460/R cells achieved with 5 and 10 µM SF was 22% and 41%, respectively (Figure 7A). SF also significantly decreased *vegf165* expression in U87-TxR cells treated with 5 and 10 µM by 48% and 45%, respectively (Figure 7B).

We also assessed the VEGF expression by flow-cytometry (Figure 7C, D, E, F). We used direct PE-labeled antibody for VEGF to study the changes in protein expression level. Significant decreases in mean VEGF fluorescence of NCI-H460/R and U87-TxR cells treated with 10 µM SF were observed after 24 h and 48 h, respectively (Figure 7C, D). The decrease of VEGF expression was transient. The best effect was illustrated by flow-cytometric profiled histogram (Figure 7E, F). The fluorescence in SF treated samples declined in comparison to untreated controls of NCI-H460/R and U87-TxR by 22.5% and 51.6%, respectively.

The secretion of VEGF_165_ protein was examined in cell culture medium (Figure 7G, H). The culture medium was collected from untreated and treated samples with the same amount of cells as well as when the amount of cells in treated samples decreased due to SF action.

The secretion of VEGF_165_ after 24 h, 48 h and 72 h by untreated U87-TxR cells (4620, 6070 and 8020 pg/µl, respectively) exceeded the secretion obtained by untreated NCI-H460/R cells (359, 845 and 1459 pg/µl, respectively). Secretion of VEGF_165_ was highly stimulated (1220, 2323 and 2629 pg/µl, respectively) when NCI-H460/R cells were treated with 10 µM SF (Figure 7G, grey bars). However, the level of secreted VEGF_165_ decreased significantly after 72 h from 1459 pg/µl in control samples to 973 pg/µl in SF treated samples if the results were not normalized (Figure 7G, black bars). U87-TxR cells also significantly elevated the level of secreted VEGF_165_ after 10 µM SF treatment, but only at 24 h (Figure 7H, grey bars). Contrary, when the levels of secretion were not normalized, significant decreases in detected VEGF_165_ were observed after 48 h and 72 h of SF treatment (Figure 7H, black bars). This could be the consequence of SF anti-proliferative and apoptotic effect as well as of significant decrease of VEGF expression observed in U87-TxR cells after 48 h of SF treatment.

According to obtained results, SF modulates the secretion of VEGF and reduces its expression and the synthesis of corresponding mRNA. Therefore, we assume that SF exerts its anti-angiogenic potential by empting the intracellular pool of VEGF and preventing the renewal of its synthesis. However, the detected elevation of secreted VEGF in medium could be the consequence of VEGF leaking from dying cells. This is supported by the fact that the level of VEGF in medium was actually decreased along with the decrease in number of viable cells after SF treatment. Further experiments, particularly in co-culture with endothelial cells, are required to clarify this point.

### Conclusions

Achieving an effective treatment of lung cancer is difficult, in particular in advanced stage diagnosed disease [42]. Physiological CNS barriers that prevent penetration of systemically delivered molecules limit treatment of brain malignancies [43]. Furthermore, resistance to standard chemotherapeutic agents presents major challenge in cancer treatment. MDR phenotype allows a cancer cell exposed to a single agent to become simultaneously resistant to both that drug and to other drugs of unrelated structure or function. P-gp, which is often involved in development of MDR, is responsible for the unidirectional efflux of drugs from cancer cells [44].

We showed that SF anti-cancer effects in resistant NSCLC and glioblastoma cell lines include depletion of GSH, reversion of MDR through inhibition of P-gp expression and activity, and modulation of VEGF intracellular pool. This mechanistic study explains SF action through modification of redox status and HIF-1α regulation and rationalize the use of SF alone or in combination with conventional anti-cancer agents. By this means, SF should be considered as drug that disturbs diverse mechanisms involved in cancer progression, beneficial for combination with other chemotherapeutics, particularly substrates for P-gp. Since SF inhibits HIF-1α-induced transcription of target genes important for chemoresistance and metastasis such as *mdr1* and *vegf*, our results may be useful for translation in clinic, especially for targeting solid tumors with hypoxic regions or highly-angiogenic tumors.
